# Synapsin E-domain is essential for α-synuclein function

**DOI:** 10.1101/2023.06.24.546170

**Published:** 2023-06-26

**Authors:** Alexandra Stavsky, Leonardo A. Parra-Rivas, Shani Tal, Kayalvizhi Madhivanan, Subhojit Roy, Daniel Gitler

**Affiliations:** 1 Department of Physiology and Cell Biology, Faculty of Health Sciences and School of Brain Sciences and Cognition, Ben-Gurion University of the Negev, Beer Sheva, Israel; 2 Department of Pathology, University of California, San Diego, 9500 Gilman Drive, La Jolla, CA, USA; 3 Aligning Science Across Parkinson’s (ASAP) Collaborative Research Network, Chevy Chase, MD 20815; 4 Department of Neurosciences, University of California, San Diego, 9500 Gilman Drive, La Jolla, CA, USA

**Keywords:** α-synuclein, synapsin, synapses, pHluorin, vesicle pools

## Abstract

The cytosolic proteins synucleins and synapsins are thought to play cooperative roles in regulating synaptic vesicle (SV) recycling, but mechanistic insight is lacking. Here we identify the synapsin E-domain as an essential functional binding-partner of α-synuclein (α-syn). Synapsin E-domain allows α-syn functionality, binds to α-syn, and is necessary and sufficient for enabling effects of α-syn at the synapse. Together with previous studies implicating the E-domain in clustering SVs, our experiments advocate a cooperative role for these two proteins in maintaining physiologic SV clusters.

## RESULTS AND DISCUSSION

Substantial evidence links the small presynaptic protein α-syn to neurodegenerative diseases, collectively called synucleinopathies. The normal function of α-syn has been investigated for over a decade, and a prevailing view is that α-syn is a physiologic attenuator of neurotransmitter release. Modest overexpression of α-syn dampens synaptic responses (1–5), and analogously, eliminating α-syn leads to phenotypes consistent with augmented synaptic release (6–11), although the latter has not been seen in all studies (12). At a cellular level, synaptic attenuation is likely mediated by effects of α-syn on vesicle organization and trafficking, which are even seen in minimal in-vitro systems, where recombinant α-syn clusters small synaptic-like vesicles (3, 13). An emerging model is that α-syn plays a role in the organization and mobilization of SVs, that in turn regulates SV-recycling and neurotransmitter release; however, underlying mechanisms are unknown.

Work over several decades has shown that temporal and spatial regulation of the SV cycle is achieved by the cooperative effort of diverse groups of proteins, such as Muncs/SNAREs – orchestrating SV docking, priming, fusion – and sequential assembly of a variety of endocytosis-related proteins that build a platform for efficient membrane retrieval. Reasoning that an understanding of functional α-syn partners would offer meaningful insight into α-syn function, we have been combining SV-recycling assays with structure-function approaches to identify the protein-network in which α-syn operates at the synapse. Using this approach, we recently found that the physiologic effects of α-syn at the synapse requires synapsins (4). While modest over-expression of α-syn in wild-type (WT) cultured hippocampal neurons attenuated SV recycling, there was no effect in neurons lacking all synapsins, indicating that synapsins were necessary to enable α-syn functionality. Reintroduction of the canonical synapsin isoform (synapsin Ia) reinstated α-syn mediated attenuation, confirming functional cooperation between α-syn and synapsins (4).

**Figure 1:**
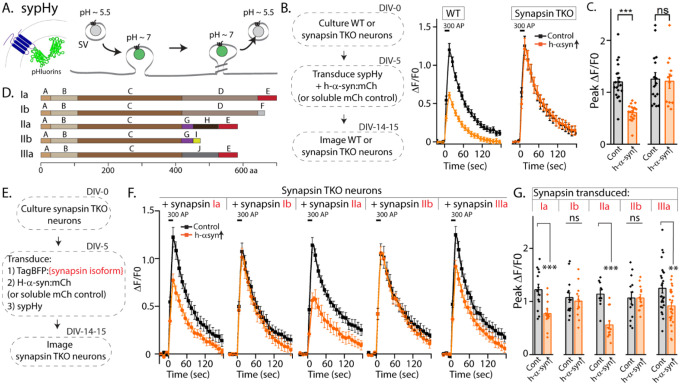
Screening for synapsin isoforms that allow α-syn functionality. **A)** Schematic showing pH-sensitive sensor sypHy and principle of pHluorin experiments to quantitatively evaluate the SV cycle (see main text and methods for more details). **B)**. Elimination of all synapsins block α-syn functionality at synapses. Left: Schematic showing design of pHluorin experiments. WT or synapsin TKO cultured hippocampal neurons were co-transduced at 5 days in-vitro (DIV) with h-α-syn:mCherry (or mCherry as control) and sypHy, and imaged at 14–15 DIV. Right: Stimulation-induced sypHy fluorescence traces (300 action potentials at 20 Hz, delivered at t=0 sec – for clarity, symbols only mark every other mean±SEM ΔF/F_0_ value in all sypHy traces). Note that while h-α-syn over-expression (orange) attenuated sypHy fluorescence in WT neurons, there was no effect in neurons from mice lacking all synapsins (TKO). All sypHy data quantified in (**C**). **C)**. Quantification of peak ΔF/F_0_ sypHy values. A total of 12–19 coverslips were analyzed for each condition, from at least 3 separate cultures (***p=1e-7, ns *p*=0.90, U-test). **D)**. Domain structure of the five main synapsin isoforms. **E)**. Experimental design to identify the synapsin isoform that reinstated α-syn functionality, Synapsin TKO neurons were co-transduced at 5 DIV with each synapsin isoform, h-α-syn, and sypHy; and imaged at 14–15 DIV. **F)**. SypHy fluorescence traces (mean±SEM). Note that h-α-syn (orange) attenuates SV recycling only if the neurons are also co-expressing the “a” isoforms – synapsins Ia, IIa and IIIa (300 action potentials at 20 Hz, delivered at t=0 sec). Data quantified in G. **G)**. Quantification of peak ΔF/F_0_ sypHy values. 13–26 coverslips from at least 3 separate cultures were analyzed for each condition (***p=0.0009, ns *p*=0.62, ***p=0.00005, ns *p*=0.62 for Ib and *p*=0.99 for IIb; **p=0.004, Student’s t test).

Synapsins are a family of cytosolic proteins with known roles in maintaining physiologic SV clusters (14, 15). Alternative splicing of three synapsin genes gives five major isoforms. Both synapsins and synucleins are peripherally associated with SVs via the N-terminus, while C-terminal regions are more variable and structurally disordered. Depending on the isoform, the C-terminus of synapsin has 2–3 structurally distinct domains, and substantial evidence indicates that this domain-variability leads to isoform-specific functions (16). Reasoning that identifying the specific synapsin domain/isoform that bound to α-syn and facilitated α-syn function would offer mechanistic insight into α-syn biology, we systematically evaluated effects of each synapsin isoform in enabling the physiologic effects of α-syn. For these experiments, we used pHluorin assays that report exo/endocytic SV recycling. The pHluorin is a pH-sensitive GFP that acts as a sensor for pH changes, and in our experiments, the probe is tagged to the transmembrane presynaptic protein synaptophysin, and targeted to the interior of SVs [called “sypHy”, see (17)]. In resting SVs, sypHy is quenched, as the pH is acidic (~ 5.5). However, upon stimulation, SVs fuse with the presynaptic plasma membrane, resulting in pH-neutralization and a concomitant rise in fluorescence, which is subsequently quenched as the vesicles are endocytosed and reacidified (Fig. 1A). Fluorescence fluctuations in this assay is a measure of SV exo/endocytosis, and at the end of the experiment, all vesicles are visualized by adding NH_4_Cl to the bath (alkalinization). As reported previously, overexpression of h-α-syn attenuated SV recycling in WT hippocampal cultured neurons, but there was no effect in neurons from mice lacking all synapsins – synapsin triple knockout or TKO mice (Fig. 1B–C).

**Figure 2:**
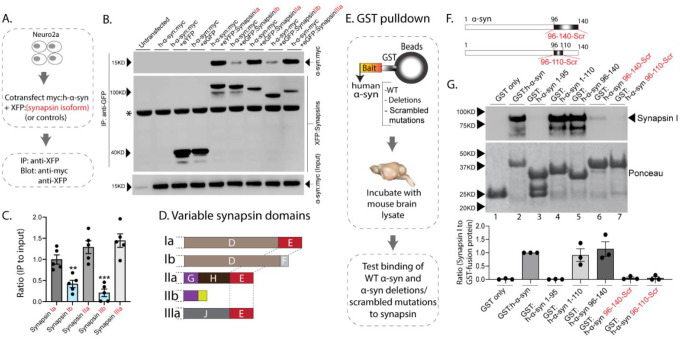
Interaction of synapsin isoforms with h-α-syn. **A)**. Workflow for co-immunoprecipitation experiments in neuro2a cells. **B)**. Western blots from co-immunoprecipitation experiments show that the synapsin isoforms Ia, IIa, and IIIa associate more robustly with h-α-syn (top panel), when compared to synapsins Ib and IIb (a non-specific band is marked with an asterisk). **C)** Quantification of blots in **(B)** n=5, all data presented as mean ± SEM (**p < 0.01, ***p < 0.001, Student’s t-test). **D)**. Schematic showing synapsin isoforms and their variable domains. Note that the E-domain is common between synapsins Ia, IIa and IIa. **E)**. Workflow for pulldown of GST-tagged h-α-syn WT/deletions/scrambled mutations after incubation with mouse brain lysates. Equivalent amounts of immobilized GST α-syn variants were used. **F)**. Schematic showing α-syn regions that were scrambled (amino acids between 96–140 and 96–110). **G)**. Top: Samples from GST-pulldown were analyzed by NuPAGE and immunoblotted with an antibody against synapsin I (top panel). Bottom: Ponceau staining shows equivalent loading of fusion proteins. Note that full-length h-α-syn bound synapsin I from mouse brains (lane 2), while deletion of the h-α-syn C-terminus (amino acids 96–140, lane 3) eliminated this interaction. Lanes 4–7 show that the sequence within amino acids 96–110 of h-α-syn is critical for binding to synapsin I. All western blots are quantified below (n=3). Data presented as mean ± SEM ***p < 0.001, Student’s t-test.

The synapsin family has five main isoforms, Ia Ib, IIa, IIb, and IIIa (Fig. 1D). To determine synapsin isoforms that enable α-syn functionality, we overexpressed h-α-syn in cultured neurons from synapsin TKO mice and systematically reintroduced each synapsin isoform, with the goal of identifying synapsin isoforms that reinstated α-syn-induced synaptic attenuation (see plan in Fig. 1E). Interestingly, only synapsins Ia, IIa and IIIa enabled h-α-syn-mediated synaptic attenuation, whereas synapsins Ib and IIb had no effect (Fig. 1F, quantified in 1G). One prediction of the pHluorin experiments is that only synapsin isoforms that allow for α-syn functionality would bind α-syn. To test this, we performed co-immunoprecipitation experiments in neuronal cell lines, where we co-transfected neuro-2a cells with myc-tagged h-α-syn and each synapsin isoform (fluorescent-tagged), immunoprecipitated the synapsin isoform, and determined amounts of immunoprecipitated α-syn by western blotting (schematic in Fig. 2A). While synapsins Ia, IIa, and IIIa bound robustly to h-α-syn, binding of synapsins Ib and IIb was much lower (Fig. 2B, quantified in Fig. 2C). Taken together, these experiments indicate that only three synapsin isoforms (Ia, IIa, and IIIa) can robustly bind to α-syn and reinstate functional effects of α-syn in this setting. Since the E-domain, within the variable C-terminus, is common to these three synapsin isoforms – and absent in the others (see domain-structure in Fig. 2D) – we reasoned that the E-domain was the bona fide α-syn binding-site, and also responsible for facilitating α-syn functionality.

In parallel experiments, we also narrowed down the reciprocal region in α-syn that bound to synapsin. Towards this, we designed GST-pulldown assays to test interaction of various h-α-syn sequences with mouse brain synapsins. In these experiments, beads with GST-tagged h-α-syn (WT, deletions and scrambled variants) were incubated with mouse brain lysates, and brain synapsins binding to α-syn were evaluated by western blotting (Fig. 2E). Figure 2F shows how the scrambled variants were designed. While synapsins bound to GST-tagged WT-h-α-syn, deletion of the C-terminus (α-syn 96–140) eliminated this interaction (Fig. 2G, lanes 1–3). Regions within amino acids 96–110 of α-syn were critical in binding synapsin, as this minimal region bound to synapsin (Fig. 2G, lanes 4–5), and scrambling the amino acids within this region – while keeping the other sequences intact – eliminated this interaction (Fig. 2G, lanes 6–7). Data from all western blots is quantified in Figure 2G – bottom. Taken together, these experiments identify amino-acids 96–110 of α-syn as the region binding to synapsin.

**Figure 3:**
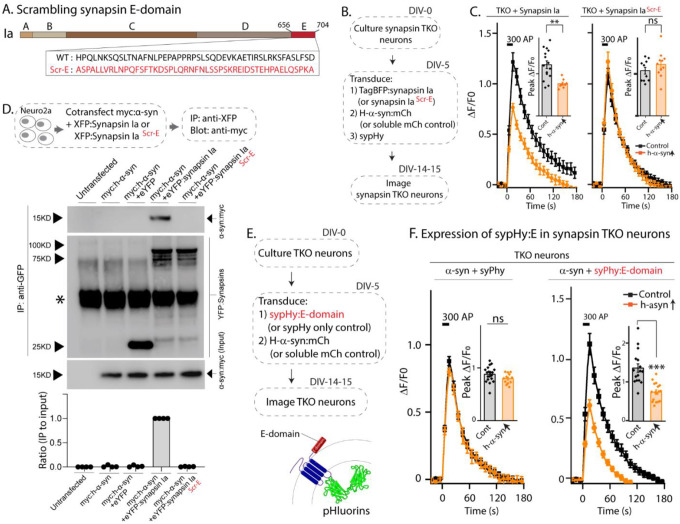
The synapsin E-domain is necessary and sufficient for enabling α-syn functionality. **A)**. Schematic showing synapsin Ia scrambled E-domain sequence (synapsin Ia^scr-E^). Numbers depict amino acid positions, letters in the inset depict amino-acids. Note that the WT amino acids are randomized in the scrambled mutant. **B)**. Design of sypHy experiments co-expressing synapsin Ia^scr-E^ and h-α-syn in cultured neurons from synapsin TKO mice. **C)**. Stimulation-induced sypHy fluorescence traces (300 action potentials at 20 Hz, delivered at t=0 sec). Note that while h-α-syn attenuated sypHy fluorescence in synapsin TKO neurons expressing synapsin Ia, h-α-syn had no effect in neurons expressing synapsin Ia^scr-E^. Insets: Quantification of peak ΔF/F_0_ sypHy values. 10–15 coverslips from at least 3 separate cultures were analyzed for each condition (**p=0.0057, one-way ANOVA with Tukey’s posthoc analysis). **D)**. Top: Schematic for co-immunoprecipitation experiments, to test the interaction of h-α-syn with WT synapsin Ia or synapsin Ia^scr-E^. Neuro2a cells were co-transfected with myc-tagged α-syn and respective YFP-tagged synapsin Ia, and the YFP was immunoprecipitated. Bottom: Note that h-α-syn co-immunoprecipitated with synapsin Ia, but not synapsin Ia^scr-E^; quantification of the gels below (n=4, all data are means ± SEM ***p < 0.001, Student’s t test – a non-specific band is marked with an asterisk). **E)**. Schematic of experiments to test if the synapsin E-domain is sufficient to enable α-syn functionality in synapsin TKO neurons. Synapsin-E (a 46 amino acid sequence) was fused to the C-terminus of sypHy, so that upon expression in neurons, the E-domain would be present on the cytosolic surface of SVs. **F)**. SypHy fluorescence traces. Note that while h-α-syn (orange) was unable to attenuate SV recycling in synapsin TKO neurons (as expected), diminished synaptic responses were seen when the E-domain was present. Insets: Quantification of peak ΔF/F_0_ sypHy values. 12–19 coverslips from at least 3 separate cultures were analyzed for each condition (***p=1.2e-7, one-way ANOVA with Tukey’s posthoc analysis).

Next, to test if the E-domain was necessary for enabling α-syn functionality, we generated a synapsin-Ia construct where the amino acid sequences of the E-domain were scrambled (Fig. 3A, synapsin-Ia^ScrE^). As shown previously, expression of WT synapsin-Ia enables α-syn-mediated synaptic attenuation in neurons lacking all synapsins (Fig. 1F, leftmost panel). We reasoned that if the E-domain enabled α-syn functions and mediated synapsin/α-syn interactions in these experiments, scrambling this region should abolish such synapsindependent functions. Towards this, we used pHluorin assays in synapsin TKO neurons, asking if synapsin-Ia^ScrE^ would fail to reinstate α-syn functionality (schematic in Fig. 3B). Indeed, while overexpressed h-α-syn was able to attenuate synaptic responses in the presence of WT-Synapsin-Ia in synapsin TKO neurons, Synapsin-Ia^ScrE^ failed to have any effect (Fig. 3C). Analogously, in neuro2a co-immunoprecipitation experiments to test binding of WT-Synapsin-Ia and Synapsin-Ia^ScrE^ to α-syn, WT h-α-syn bound to Synapsin-Ia, but not to Synapsin-Ia^ScrE^ (Fig. 3D), indicating that the E-domain is critical in mediating this interaction. Next, we tested if the synapsin-E domain was sufficient for enabling α-syn functionality. Towards this, we fused the E-domain to the C-terminus of sypHy, so that the small synapsin fragment would be localized to the cytosolic surface of SVs (Fig. 3E) – where synapsin molecules would normally localize – reasoning that placing this domain in the right cellular context may be sufficient for enabling α-syn function. Indeed, forced targeting of the synapsin E-domain to the surface of SVs restored α-syn mediated synaptic attenuation in synapsin null neurons (Fig. 3F), suggesting that the E-domain was sufficient to reinstate the functional interplay between α-syn and synapsins.

Precise organization of vesicles at synapses is critical for synaptic function (18). Typically, each synapse has clusters of dozens to hundreds of SVs, and these vesicles are classified into different pools based on their ability to participate in exocytosis, and their physical proximity to the site of exocytosis (active zone). SVs within the readily-releasable pool are docked at the active zone and can rapidly fuse with the plasma membrane in response to an action potential. On the other hand, SVs within the much larger reserve pool are distal to the active zone and are thought to help replenish SVs following exocytosis. Actively recycling vesicles comprise the recycling pool. Studies over several decades have shown that functional perturbation of synapsins selectively reduces the number of SVs in the reserve pool, establishing a role for synapsin in maintaining SV clusters within this pool [reviewed in (14)]. Previous studies have also explored the role of the E-domain in various model systems. Microinjecting domain-E antibodies into lamprey giant axons dispersed the distal cluster of SVs (19), suggesting that this domain has a role in organizing the reserve pool. Injection of a peptide from the E-domain into squid giant synapses also dispersed the distal SV cluster, while docked SVs remained intact (20), indicating that interfering with this domain in different ways resulted in the same phenotype – disruption of the reserve pool of SVs.

Thus the current view is that the synapsin E-domain has an important role in maintaining the distal reserve pool SV clusters, though this domain has other independent roles in SV exocytosis that are not well defined (16). In this context, α-syn has also been long thought to play roles in SV organization and trafficking. First, the N-terminus of α-syn adopts a helical structure in the presence of small synaptic-like vesicles (21), and can also directly modulate vesicle shape (22). In cell free systems, recombinant α-syn can cluster synaptic-like vesicles (3, 13), and experiments with cultured neurons also support that idea that α-syn can cluster SVs (2). For example, induced multimerization of α-syn at synapses clusters synaptic vesicles (2), and α-syn overexpression also diminished vesicle trafficking between synaptic boutons (23), which may reflect clustering of SVs by α-syn. Adjacent vesicles may also be directly tethered by α-syn (24, 25), thus α-syn-dependent organization and corralling of SVs are important clues to its function. Interestingly, a recent study showed that injection of an antibody to the N-terminus of α-syn into lamprey giant axons also led to a loss of SVs (26) – resembling the SV disruption caused by synapsin E-domain injections (19, 20) – though both reserve and readily-releasable pools were depleted with α-syn injections. Our studies open the door to further mechanistic investigations into the functional interacting partners of α-syn, which will be important to uncover the myriad functions of this enigmatic protein. More broadly, our structure-function experiments places α-syn in a functional context with its interacting partners at the synapse, offering new insight into α-syn biology.

## Supplementary Material

Supplement 1
