# Supplementary material for: Synapsin E-domain is essential for α-synuclein function
Source: bioRxiv. 2023 Dec 7:2023.06.24.546170. Originally published 2023 Jun 26. Preprint. [Version 2] doi: 10.1101/2023.06.24.546170 (PMC10327093; doi:10.1101/2023.06.24.546170)
Supplement: Supplement 1 [file NIHPP2023.06.24.546170V2-supplement-1.pdf]

bioRxiv preprint doi: https://doi.org/10.1101/2023.06.24.546170; this version posted December 7, 2023. The copyright holder for this preprint (which was not certified by peer review) is the author/funder, who has granted bioRxiv a license to display the preprint in perpetuity. It is made available under aCC-BY 4.0 International license.

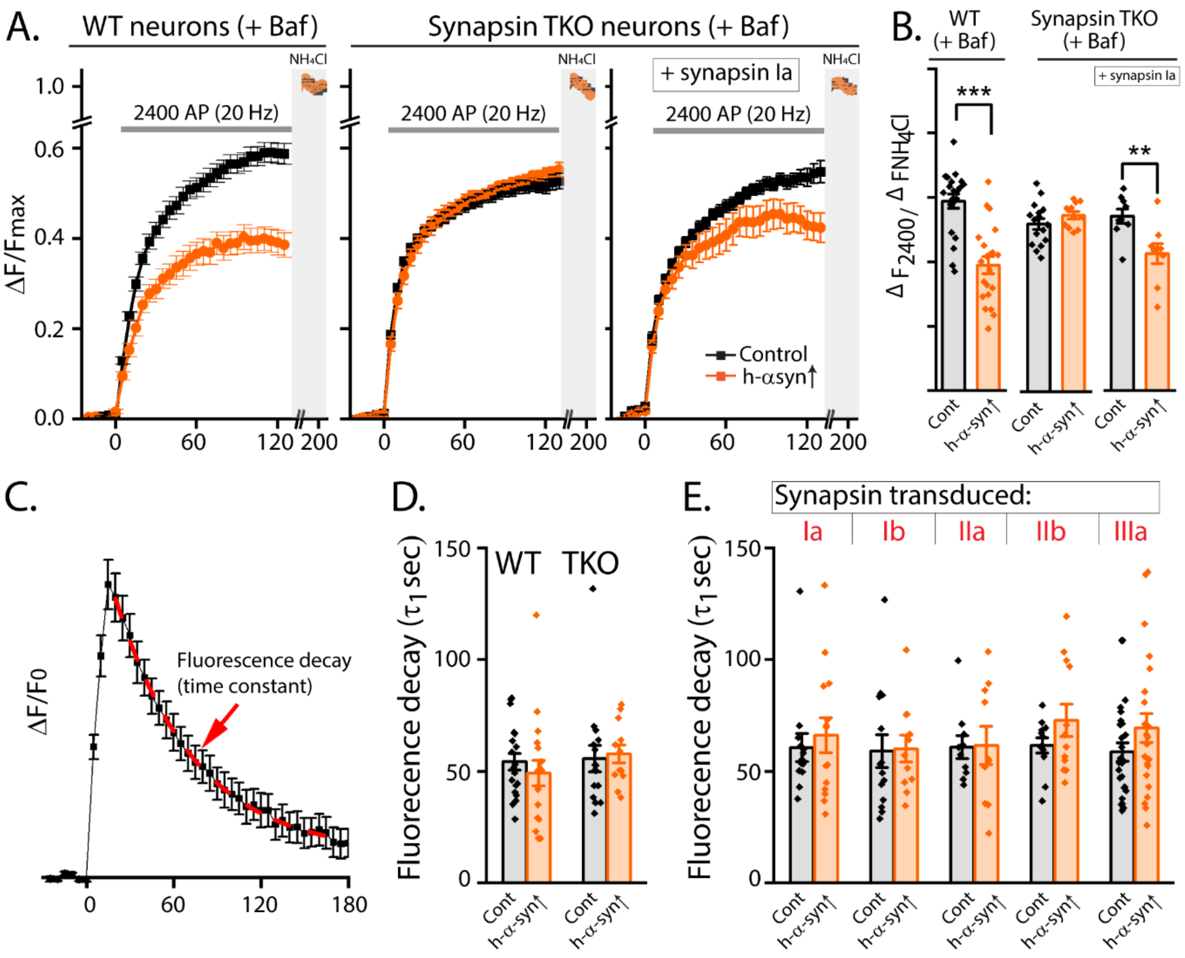

<u>Figure 1 - figure supplement 1</u>: Effects of h-α-syn over-expression are largely due to suppression of exocytosis.

A) Data from sypHy experiments where reacidification was blocked by bafilomycin (Baf), allowing isolated evaluation of exocytosis only (also see results). Note that h-α-syn over-expression attenuated synaptic exocytosis in WT neurons (left), while there was no effect in synapsin TKO neurons (middle). Reintroduction of tagBFP:synapsin Ia reinstated the h-α-syn mediated synaptic attenuation (right). All pHluorin data quantified in **(B)**. 9 to 22 coverslips from at least 3 independent cultures (****p*=1.9e-5 Mann-Whitney test, *p*=0.22 Student's t-test, ***p*=0.008 Student's t-test).

C) A representative trace showing how the fluorescence decay was quantified to evaluate endocytosis in the sypHy experiments (also see results).

D-E) Fluorescence decay analyses of h-α-syn over-expression in WT and synapsin TKO neurons (D), as well as in synapsin TKO neurons where each synapsin isoform was reintroduced (E). Note that there were no significant differences in any of these groups. All data in this figure are represented as mean +/- SEM. 10 to 26 coverslips from at least 3 independent cultures (D: *p*=0.46; E: *p*=0.85 both Kruskal Wallis ANOVA).

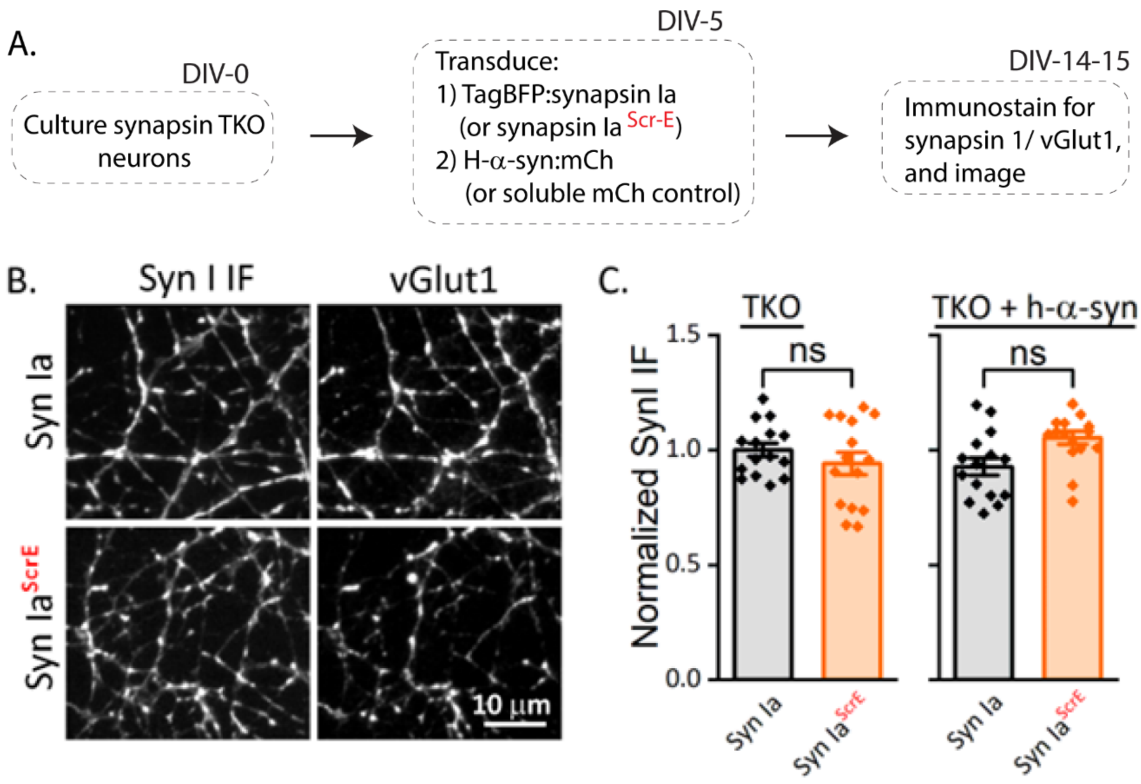

<u>Figure 3-figure supplement 1</u>: Similar synaptic of localization of synapsin Ia and synapsin Ia^{Scr-E} in synapsin TKO neurons.

A) Schematic of experiments to evaluate quantitative localization of tagBFP:synapsin Ia and tagBFP:synapsin Ia^{Scr-E} in synapsin TKO neurons (with and without h-α-syn over-expression). Neurons were immunostained for synapsin I (for reliable visualization of the transduced synapsin constructs), as well as for the SV-marker vGlut1 (to confidently identify synapses).

B) Representative images showing equivalent immunofluorescence of synapsin Ia and synapsin Ia^{Scr-E} at synapses. Over-expression of h-α-syn did not affect their synaptic fluorescence.

C) Quantified synaptic fluorescence data, represented as mean +/- SEM, 15-16 coverslips from at least 3 independent cultures were analyzed for each condition. ns p=0.52, ns p=0.14, one-way ANOVA.

bioRxiv preprint doi: https://doi.org/10.1101/2023.06.24.546170; this version posted December 7, 2023. The copyright holder for this preprint (which was not certified by peer review) is the author/funder, who has granted bioRxiv a license to display the preprint in perpetuity. It is made available under aCC-BY 4.0 International license.

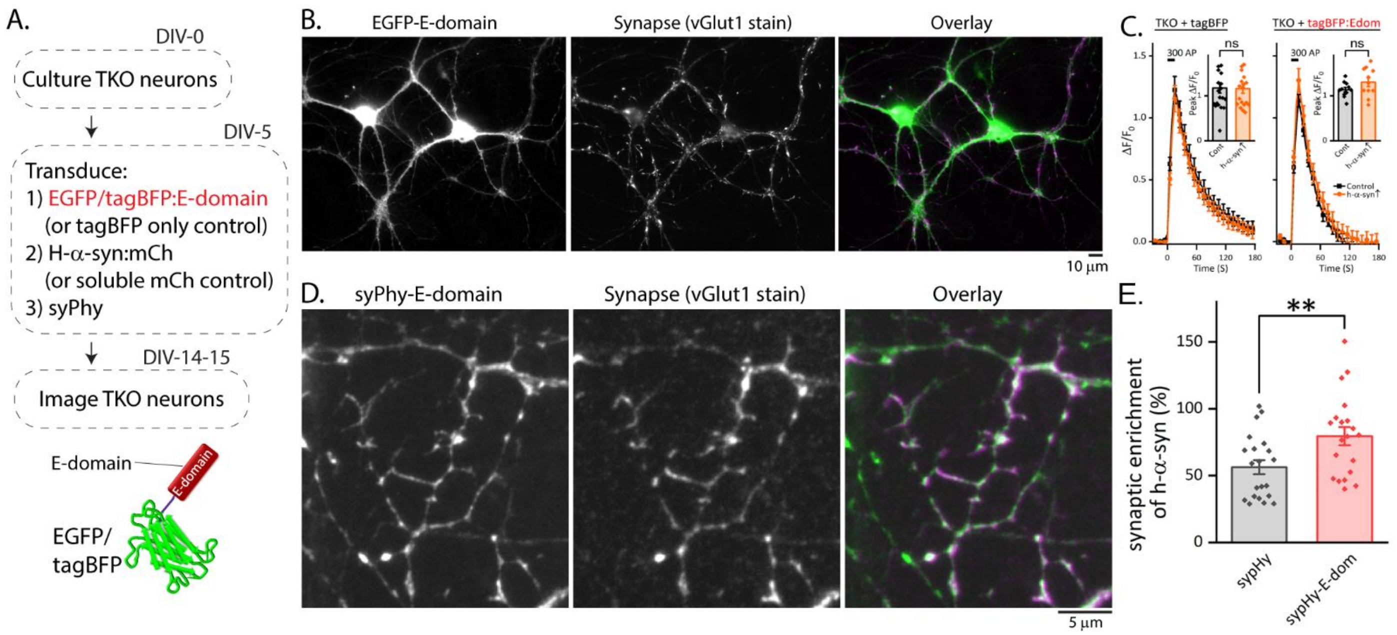

Figure 3-figure supplement 2: Synaptic targeting of synapsin E-domain constructs in synapsin null neurons.

A) Schematic of experiments to evaluate synaptic targeting of synapsin E-domain constructs in synapsin TKO neurons. Note that EGFP is tagged to the E-domain in these experiments.

B) The EGFP:E-domain construct was diffusely distributed in neurons and not enriched to synapses (marked by immunostaining of VGlut1). A representative image showing that the E-domain construct is not targeted to synapses (green: EGFP:E-domain, magenta: vGlut1).

C) Over-expression of synapsin E-domain in the context of excessive α-syn did not have any effect on SV recycling (as determined by sypHy experiments), presumably because the E-domain fails to enrich at synapses. 11-20 coverslips from at least 3 independent cultures were analyzed for each condition (ns p=0.99, ns p=0.79, one-way ANOVA with Tukey's posthoc analysis).

D) Representative images illustrating synaptic localization of the E-domain tagged to sypHy (green: sypHy:E-domain, magenta: vGlut1).

E) Expression of sypHy:E-domain in synapsin TKO neurons enhances the synaptic enrichment of h-α-syn. Synaptic enrichment (see methods section) of h-α-syn was measured in synapsin TKO neurons expressing either sypHy or sypHy-E-domain. We observed significantly higher enrichment of h-α-syn in the latter. 23 to 25 coverslips from 3 independent cultures were analyzed for each condition (**p=0.009, Mann-Whitney U-test).
