# Supplementary material for: Synapsin E-domain is essential for α-synuclein function
Source: bioRxiv. 2023 Jun 26:2023.06.24.546170. Preprint. [Version 1] doi: 10.1101/2023.06.24.546170 (PMC10327093; doi:10.1101/2023.06.24.546170)
Supplement: Supplementary file 1 [file media-1.pdf]

Supplementary Methods

Animals, antibodies, and DNA constructs

All animal studies were performed in accordance with the guidelines of the Ben-Gurion University Institutional Committee for Ethical Care and Use of Animals in Research (protocol IL-52-07-2019A) and UCSD. Synapsin triple knock-out (TKO) mice were backcrossed onto the C57BL6 background as described previously (Shulman et al 2015, Gitler et al 2004). The following antibodies were used for immunofluorescence experiments: goat anti-Vglut1 (Synaptic system #135307, 1:1000), donkey anti-goat IgG NL-637 (R&D Systems #NL002, 1:1000), VAMP2 (Synaptic systems #104211, 1:1000). The following antibodies were used for biochemistry experiments: synapsin-1 (Abcam #ab254349), c-myc (Sigma #M4439, 1:500), GFP (Abcam#ab290, 1:5000). cDNAs of tagged synapsin isoforms, E domain variants, and fluorescent sensors [TagBFP:Synapsin-Ia/Ib/IIa/IIb/IIIa (Gitler et. al 2004), TagBFP:Synapsin Ia^{ScrE}, TagBFP:E-domain, h-α-syn:mCherry, synaptophysinI-2XpHluorin (sypHy) and sypHy:E-domain] were obtained by PCR or digestion of existing plasmids and subcloned into an adeno-associated virus (AAV) backbone that contains the human synapsin promoter, the woodchuck post-transcriptional regulatory element (WPRE) and the bovine growth hormone polyadenlynation signal (bGHpA) (Kugler et al. 2003). XFP tagged synapsin and the E-domain were previously described (Gitler et al. 2004). The sequence of the synapsin Ia E-domain was scrambled using the online tool Peptide Nexus (https://peptidenexus.com/article/sequence-scrambler). A synthetic DNA block (IDT) coding for the scrambled E domain was subcloned using Gibson-cloning (NEB). GST-α-syn 96-140 Scr and GST-α-syn 96-110 Scr plasmids were synthesized by GeneScript. Constructs obtained from other laboratories are noted in the acknowledgments. All constructs were verified by sequencing.

Hippocampal Cultures, AAV production, and transduction

Primary hippocampal cultures were obtained using standard procedures as described previously (Tevet et al. 2016; Stavsky et al. 2021). In brief, P0-P2 pups of either sex were decapitated, and the brains were quickly removed. Dissected hippocampi were kept on ice in Hank's Balanced Salt Solution (HBSS, Biological Industries) supplemented with 20mM HEPES at pH 7.4. Hippocampus pieces were incubated for 20 minutes at room temperature (RT) in a digestion solution consisting of HBSS, 1.5mM $CaCl_2$, 0.5mM EDTA, and 100 units of papain (Worthington) activated with cysteine (Merck). The brain fragments were then triturated gently two times using fire-polished glass pipettes of decreasing diameter. Cells were seeded at a density of 80,000-100,000 cells per well on glass coverslips coated with poly-D-Lysine (Merck). Cells were plated in Neurobasal-A medium supplemented with 2% B27, 2 mM Glutamax I (Thermo-Fisher Scientific), 5% FBS and 1 µg/ml gentamicin (Biological Industries). After 24 hours, the medium was replaced with serum-free medium containing of Neurobasal-A, 2 mM Glutamax I, and 2% B27. Cultures were maintained at 37°C in a 5% CO_2 humidified incubator until used. For AAV production, HEK293T cells were co-transfected with the targeting plasmid and two helper plasmids (pD1 and pD2). Hybrid AAV1/2 viral particles were produced as described previously (Tevet et al 2016). Neurons were transduced at 5-6 DIV by the addition of the viral particles to the growth medium and incubated for at least 7 days before imaging. Viral titers were individually adjusted to produce ~90% transduction efficiency. Expressed proteins were verified by western blot and immuno-labeling analysis.

Phluorin essays, analysis, and fluorescence microscopy

Neurons expressing sypHy were imaged at 12-14 DIV. Experiments were conducted in standard extracellular solution containing (in mM): NaCl 150, KCl 3, Glucose 20, HEPES 10, $CaCl_2$ 2, $MgCl_2$ 3, pH adjusted to 7.35. To block recurrent network activity, experiments were conducted in the presence of 10µM DNQX (6,7-Dinitroquinoxaline-2,3 (1H,4H-dione, Merck) and 50µM APV (DL-2-Amino-5-phosphonopentanoic acid, Merck). At the completion of each experiment, the bath was perfused with saline in which 50 mM NaCl was replaced with NH_4Cl to visualize the total vesicle population. For imaging, cultured neurons were placed in a stimulation chamber between parallel platinum wires (RC-49MFSH, Warner Instruments). Stimulation (300 bipolar pulses of 10 V/cm, each of a duration of 1 µs, at 20 Hz for 15 s), was delivered using a high-power stimulus isolation unit (SIU-102B, Warner Instruments) driven by an isolated pulse stimulator (2100, A-M Systems). 50 images were obtained (43 at 0.2 Hz and then 7 images at 0.125 Hz) per experiment. At least 30 synaptic regions of interest (ROIs) were analyzed in each case. The baseline sypHy fluorescence (F_0) in each synapse was the average value measured in 5 pre-stimulation images. The fluorescence increment at time t [$\Delta F(t)=F(t)-F_0$] was normalized by the baseline value for each synapse. Synaptic $\Delta F(t)/F_0$ values were averaged across synapses in each experiment (shown as symbols in bar-chart graphs). These were averaged to obtain mean values for each experimental condition. Non-responding synaptic puncta were excluded. Experiments were performed using at

least three independent cultures on different days. To confirm the presence of h-α-syn-mCherry and tagBFP-synapsins fluorescent-tagged proteins were imaged before each experiment. All pHluorin assays were performed at room temperature. Fluorescence measurements were performed on a Nikon TiE inverted microscope driven by the NIS-elements software package (Nikon). The microscope was equipped with an Andor Neo 5.5 sCMOS camera (Oxford Instruments), a 40X 0.75 NA Plan Fluor objective, EGFP and Cy3 filter cubes (TE-series, Chroma), BFP, mCherry and Cy5 filter cubes (Semrock), and a perfect-focus mechanism (Nikon).

Biochemical assays and evaluation

Preparation of brain and neuro2A lysates

Whole mouse brains were homogenized with a Dounce tissue grinder in neuronal protein extraction reagent (N-PER) (Thermo Scientific) containing protease/phosphatase inhibitors (Cell Signaling #5872). Triton X-100 was added to a final concentration of 1%, and the samples were incubated with rotation for 1 hour at 4 °C. Samples were centrifuged at 10,000 × *g* for 10 min at 4 °C, and the supernatant was collected. To obtain Neuro2A lysates, cells were washed with 1X PBS 3 times and incubated for 5 min on ice in the presence of N-PER reagent supplemented with protease inhibitors. Samples were centrifuged at 10,000 × *g* for 10 min at 4 °C to remove cellular debris. After obtaining the brain and Neuro2A lysates, we measured protein concentration (DC™ Protein Assay Kit II, Biorad), and samples were used in subsequent experiments.

Immunoprecipitations and Western blots analysis

Immunoprecipitations were performed using 1-2 mg of total protein. Samples were incubated overnight with the indicated antibody at 4 °C, followed by the addition of 50 µl of protein G-agarose beads (Thermo Scientific). Immunoprecipitated proteins were recovered by centrifugation at 2,500× rpm for 2 min, washed three times with a buffer containing PBS and 0.15% Triton X-100. The resulting pellets were resuspended in 20 µl of 1X NuPAGE LDS sample buffer (Thermo Scientific #NP007) and incubated at 95 °C for 10 min. Samples were separated by NuPAGE 4 to 12% Bis-Tris polyacrylamide gels (Thermo Scientific #NP0335BOX), and transferred to a 0.2 µM PVDF membrane (Thermo Scientific #LC2002), using the Mini Blot Module system (Thermo Scientific). PVDF membranes were first fixed with 0.2% PFA 1x PBS per 30min at room temperature. Then, membranes were washed three times for 10 min in PBS with 0.1% Tween 20 Detergent (TBST) and blocked for 1 h in TBST buffer containing 5% dry milk, and then incubated with the indicated primary antibody for 1 h in blocking solution, washed three times for 10 min each, and incubated with an HRP-conjugated secondary antibody. After antibody incubations, membranes were again washed three times with TTBS buffer and protein bands were visualized using the ChemiDoc Imaging System (BioRad) and quantified with Image Lab software (BioRad).

GST fusion proteins production

Full-length recombinant human WT α-syn, α-syn 1-95, α-syn 1-110, α-syn 96-140, α-syn 96-140 Scr and α-syn 96-110 Scr were expressed in *Escherichia coli* BL21 (DE3) (New England Biolab, Cat # C2530H) using the bacterial expression vector pGEX-KG. Following transformation protein expression was induced with 0.05 mM IPTG (isopropyl-β-d-thiogalactopyranoside), and, either incubated at 37°C for 2 hours, or at room temperature for 6 hours, with shaking. The cells grown on Terrific Broth (Thermo Scientific Cat# BP9728-2) were harvested by centrifugation at 4500× *g* at 4 °C for 20 min and pellets were stored at -80 °C until use. For protein purification, protein pellets were resuspended in 30 ml Lysis Buffer containing 1X PBS, 0.5 mg/ml lysozyme, 1 mM PMSF, DNase, and EDTA-free protease cocktail inhibitor (Roche Cat# 11836170001) for 15 min on ice, briefly sonicated (3 sets with 33 strikes and 30 second breaks on ice between sets), and removed the insoluble material by centrifugation at 15,000g at 4 °C for 30 min. The clarified lysate was incubated with 500 µl of glutathione-Sepharose 4B (Sigma Cat# 17-0756-01), preequilibrated with 1X PBS containing 0.1% Tween 20 and 5% glycerol (binding buffer), on a tumbler at 4°C overnight. The GST-bound proteins were washed four times with 30 ml binding buffer and maintained at 4°C for pull-down assays.

Pull-down Assays

To pull down Synapsin Ia from brain lysates, 1-2 mg of the sample was incubated with 25-50 µg of glutathione beads containing GST fusion proteins for 12-16 hrs. The mixtures were washed three times with 1X PBS with 0.15% Triton X-100, and then resuspended in 20 µl of 1X NuPAGE LDS sample buffer for NuPAGE and immunoblotted analysis.

Statistical Analysis

Bar chart values represent mean±SEM values, and symbols are the results of individual experiments. Normality of the distribution was tested using the Shapiro-Wilk test. Pairs of datasets were compared using the two-sided Students' t-test when deemed to be normally-distributed, otherwise using the Mann-Whitney's non-parametric u-test. Multiple comparisons of normally distributed datasets were performed using either one-way ANOVA followed by Dunnett's-test post-hoc analysis or using two-way ANOVA, followed by Tukey's post-hoc analysis. Outliers were identified using Grubbs's test. Statistical significance was set at a confidence level of 0.05 for all tests. In all figures: "ns" denotes $p>=0.05$; * $p<0.05$; ** $p<0.01$; and *** $p<0.001$. Statistical analysis was performed using Origin 2022 (Originlab) or Prism 6 software (GraphPad).

SUPPLEMENTARY REFERENCES

1. Shulman, Y. *et al.* ATP Binding to Synaspsin IIa Regulates Usage and Clustering of Vesicles in Terminals of Hippocampal Neurons. *J. Neurosci.* **35**, 985–98 (2015).

2. Gitler, D. *et al.* Molecular determinants of synapsin targeting to presynaptic terminals. *J. Neurosci.* **24**, 3711–20 (2004).

3. Tevet, Y. & Gitler, D. Using FRAP or FRAPA to Visualize the Movement of Fluorescently Labeled Proteins or Cellular Organelles in Live Cultured Neurons Transformed with Adeno-Associated Viruses. in *Methods in molecular biology (Clifton, N.J.)* (eds. Schwartzbach, S. D., Skalli, O. & Schikorski, T.) vol. 1474 125–151 (Springer New York, 2016).

4. Stavsky, A. *et al.* Aberrant activity of mitochondrial NCLX is linked to impaired synaptic transmission and is associated with mental retardation. *Commun. Biol.* **4**, (2021).

5. Kügler, S., Kilic, E. & Bähr, M. Human synapsin 1 gene promoter confers highly neuron-specific long-term transgene expression from an adenoviral vector in the adult rat brain depending on the transduced area. *Gene Ther.* **10**, 337–347 (2003).
